# Exendin-4 enhances the migration of adipose-derived stem cells to neonatal rat ventricular cardiomyocyte-derived conditioned medium via the phosphoinositide 3-kinase/Akt-stromal cell-derived factor-1α/CXC chemokine receptor 4 pathway

**DOI:** 10.3892/mmr.2015.3243

**Published:** 2015-01-22

**Authors:** HAO ZHOU, JUNJIE YANG, TING XIN, TAO ZHANG, SHUNYIN HU, SHANSHAN ZHOU, GUANGHUI CHEN, YUNDAI CHEN

**Affiliations:** 1 Department of Cardiology, Chinese People’s Liberty Army General Hospital, Beijing 100853, P.R. China; 2 Department of Cardiology, Tianjin First Central Hospital, Tianjin 300192, P.R. China

**Keywords:** Exendin-4, adipose-derived stem cells, cardiomyocytes, stromal cell-derived factor-1α, CXC chemokine receptor 4, phosphoinositide 3-kinase/Akt pathways, migration

## Abstract

Adipose-derived stem cells (ADSCs) are considered a suitable source of cells for the repair of tissue following acute myocardial infarction (AMI); however, the transplantation efficiency of ADSCs remains low. Therefore, identification of an efficient method to enhance the migration of engrafted cells to the target site is required. The present study used exendin-4 (Ex-4), a glucagon-like peptide-1 receptor agonist, to optimize the migratory capacity of ADSCs. The aim was to determine the effect and mechanisms of Ex-4 on the migration of ADSCs to neonatal rat ventricular cardiomyocyte-derived conditioned medium (NRVC-CM). The ADSCs and cardiomyocytes were cultured *in vitro*. Following incubation of the ADSCs with Ex-4, cell proliferation was measured using an MTT assay and the expression levels of CXC chemokine receptor 4 (CXCR4) were investigated by reverse transctiption quantitative polymerase chain reaction (RT-qPCR), western blot analysis and flow cytometry. In addition, the expression levels of stromal cell-derived factor-1α (SDF-1α) were evaluated in the NRVC-CM treated with Ex-4 by ELISA, RT-qPCR and western blot analysis. The migration of the ADSCs to the NRVC-CM was examined using a Transwell assay. Changes in the protein expression levels of phosphorylated (p−)Akt were examined in the two types of cell by western blot analysis. The results suggested that Ex-4 promoted the proliferation and expression of CXCR4 in the ADSCs, increased the secretion of SDF-1α in the cardiomyocytes and increased the expression levels of p-Akt in both cells. However, the alterations to the SDF-1α/C XC R4 cascade in the cells were abrogated following pretreatment with LY-294002, a phosphoinositide 3-kinase(PI3K) inhibitor. Furthermore, a Transwell migration assay revealed marked translocation of the ADSCs through the membranes, towards the NRVC-CM, following treatment with Ex-4. However, these effects were reduced significantly by pretreatment of the cells with the SDF-1α/CXCR4 cascade antagonist, AMD3100, and the PI3K inhibitor, LY-294002. These results indicated that Ex-4 augmented the SDF-1α/CXCR4 cascade by activating the PI3K/Akt pathways in the ADSCs and NRVCs. Furthermore, enhancement of the PI3K/Akt-SDF-1α/CXCR4 pathway may be important in the migratory response of ADSCs to NRVC-CM *in vitro*.

## Introduction

Despite major advances in the treatment of coronary artery disease, acute myocardial infarction (AMI) remains the leading cause of human mortality worldwide (1). AMI is characterized by a sudden reduction in blood and oxygen supply to the heart, irreversible muscle damage and cardiomyocyte death, resulting in the formation of an infarct zone containing nonfunctional myocytes, which are remodeled into scar tissue (2). The limited ability of the damaged heart to regenerate and replace the damaged myocardium leads to the progression of cardiac decompensation and heart failure. The development of cell-based therapeutic strategies has focused on repairing damaged vascular and cardiac tissue (2). Adipose-derived mesenchymal stem cells (ADSCs) are advantageous for therapeutic approaches due to their ease of acquirement and low levels of immunosuppression (3,4). Although the majority of animal and preliminary human studies involving the transplantation of ADSCs have exhibited an overall improvement in cardiac function (5,6), several significant problems remain, which limit the use of ADSCs in the treatment of AMI, including low rates of cell homing, retention and survival in the injured myocardium (7). The migration and homing of ADSCs to the ischemic site is a prerequisite and is crucial for myocardial repair following transplantation (8). The stromal cell-derived factor-1α (SDF-1α) chemokine and its unique receptor, CXC chemokine receptor 4 (CXCR4), are important in the mobilization and recruitment of ADSCs (9). A previous study demonstrated that SDF-1α, secreted following tissue damage induced the migration, homing and retention of CXCR4^+^ cells to the ischemic area (10). Of note, the increased expression of SDF-1α/CXCR4 leads to increased homing of transplanted stem cells to the infarcted myocardium and improves ventricular function (11). These previous findings suggest that the SDF-1α/CXCR4 cascade may be an important therapeutic target of cell‑based therapy following AMI. Therefore, there is a focus on identifying ways to regulate the expression of SDF-1α/CXCR4 (12–14).

Exendin-4 (Ex-4) is a glucagon-like peptide-1 receptor agonist, which has cell protective effects, including the inhibition of cardiomyocyte apoptosis (15), promoting the proliferation and regeneration of β-cells (16) and increasing the migration of human umbilical vein endothelial cells, observed in *in vitro* scratch wound assays (17). However, the effects of Ex-4 on the SDF-1α/CXCR4 cascade and the migration of ADSCs remain to be elucidated.

The present study aimed to investigate whether Ex-4 affected the migration of ADSCs to conditioned medium, derived from neonatal rat ventricular cardiomyocytes (NRVC-CM), via the SDF-1α/CXCR4 cascade.

## Materials and methods

### Ethics

The present study was performed in accordance with the Declaration of Helsinki and the guidelines of the Ethical Committee of the Chinese People’s Liberty Army (PLA) General Hospital (Beijing, China).

### Isolation, culture and characterization of ADSC and NRVCs

The ADSCs used in the present study were obtained from a total of five male Sprague-Dawley rats (60–80 g) obtained from the Laboratory Animal Center, Chinese PLA General Hospital, as described previously, with minor modifications, including the adjustment of collagenase I concentration to 0.1% (v/v) and the dose of trypsin to 0.05% (v/v) (18). Rats were sacrificed by cervical dislocation immediately following obtainment. Briefly, the white adipose tissue from the inguinal region was washed three times with phosphate-buffered saline (PBS; Sigma-Aldrich, St. Louis, MO, USA) and digested using collagenase I (5 ml; Sigma-Aldrich) and trypsin (5 ml; Sigma-Aldrich) at 37°C for 40–45 min with continuous agitation at 100 rpm using a MYP11–2 magnetic stirrer (Shanghai Wei Ling Scientific Instrument Co., Ltd., Shanghai, China). The digested tissue was filtered through *70-μ*m filters (BD Biosciences, Franklin Lakes, NJ, USA), followed by centrifugation for 10 min at 400 × g and resuspended in L-Dulbecco’s modified Eagle’s medium (DMEM; Gibco Life Technologies, Carlsbad, CA, USA) supplemented with 10% fetal bovine serum (Hyclone Laboratories, Inc., Logan, UT, USA), 100 units/ml penicillin and 100 mg/ml streptomycin (Sigma-Aldrich). The cell fraction was cultured at 37°C in 5% CO_2_ and the medium was refreshed every three days. The subsequent experiments were performed with ADSCs between the fourth and fifth passages.

The NRVCs were isolated by enzymatic dissociation, where the heart tissue was treated with 0.1% (w/v) collagenase for 20 min at 37°C, and then incubated with 0.25% (w/v) trypsin overnight at 4°C. The cells were subsequently plated at a density of 5×10^5^ cells/ml in DMEM supplemented with 15% (v/v) fetal calf serum (Hyclone Laboratories, Inc.) at 37°C and 5% (v/v) CO_2_, and cultured according to a standard procedure, as previously described (19). The NRVCs were maintained in H-DMEM (Gibco Life Technologies) supplemented with 20% FBS and antibiotics (100 U/ml^−1^ penicillin and 100 mg/ml^−1^ streptomycin) at a density of 2×10^4^ cells/cm^−2^ in a humidified incubator. After 48 h incubation, the cardiomyocytes were confluent and were beating spontaneously. The morphological characteristics of ADSCs and NRVCs were evaluated by inverted microscope (magnification, ×40; BX51; Olympus Corp., Tokyo, Japan).

### Flow cytometry

The ADSCs were harvested in the fourth passage to detect surface antigens. Prior to immunostaining, the cells were washed twice with PBS. The cells were then incubated with anti-rat fluorescein isothiocyanate (FITC)-labeled monoclonal antibodies CD29 (1:500; 561796; BD Biosciences), CD31 (1:200; bs-0468R-FITC; Bioss Inc., Woburn, MA, USA), CD34 (1:200; bs-2038R-FITC; Bioss Inc.), CD45 (1:500; 561867; BD Biosciences) and CD90 (1:200; bs-0778R-FITC; Bioss Inc.) at concentrations specified by the manufacturer. FITC-labeled immunoglobulin(Ig)G-stained cells were used as negative controls.

Following treatment with various concentrations of Ex-4 (0, 1, 5, 10 and 20 nm/l; Sigma-Aldrich) for 24 h, the ADSCs at passage 4 were used to detect the expression of CXCR4. The cells were washed with PBS and each group of cells was stained with either FITC-labeled CXCR4 (Bioss, Inc., Woburn, MA, USA) or FITC-labeled IgG for 30 min at room temperature. Flow cytometric analyses were performed using a BD FACSCalibur™ flow cytometer (BD Biosciences), as previously described (3).

### MTT proliferation assay

An MTT assay was performed to examine cell proliferation. ADSCs were seeded in a 96-well plate at a density of 1×10^3^ cells/well and treated with various concentrations of Ex-4 (0–20 nm/l) in triplicate for seven days at 37°C, with 5% CO_2_. Each day, 20 *μ*l MTT (5 mg/ml PBS; pH7.4; Sigma-Aldrich) was added to the cells for 4 h. The supernatants were then discarded and 100 *μ*l dimethyl sulfoxide (Sigma-Aldrich) was added to each well for 10 min. The optical density (OD) of the samples was measured at an absorbance of 490 nm (Epoch 2; BioTek Instruments, Inc., Winooski, VT, USA). The assay was repeated three times.

### Western blot analysis

The cells were washed with ice-cold PBS and lysed in radioimmunoprecipitation assay lysis buffer (Beyotime Institute of Biotechnology, Haimen, China) supplemented with protease inhibitor phenylmethanesulfonyl fluoride (1 nM/l; Beyotime Institute of Biotechnology). The proteins (60–80 μg) were separated by 10% SDS-PAGE and transferred onto polyvinylidene difluoride membranes (EMD Millipore, Billerica, MA, USA). The membranes were then blocked with tris-buffered saline plus 0.1% Tween^®^ 20 (TBST; Sigma-Aldrich) with 5% non-fat milk for 90 min at room temperature. Following blocking, the membranes were incubated with the following primary antibodies: Polyclonal CXCR4 (Santa Cruz Biotechnology, Inc., Dallas, TX, USA; 1:250), total Akt, (Santa Cruz Biotechnology, Inc.; 1:1,000), phosphorylated (p-)Akt (Santa Cruz Biotechnology, Inc.; 1:1,000), SDF-1α, (Abcam, Cambridge, MA, USA; 1:1,000) and β-actin (Santa-Cruz Biotechnology, Inc.; 1:2,000) in TBST containing 5% non-fat milk at 4°C overnight. The membranes were then washed with TBST and incubated for 1 h with horseradish peroxidase-conjugated secondary antibodies. The blots were visualized using enhanced chemiluminescence reagents (BeyoECL Plus; Beyotime Institute of Biotechnology). The mean densities of the bands were represented as the OD in units per square millimeter and normalized to that of β-actin (Quantity One, version 4.6.2; Bio-Rad Laboratories, Inc., Hercules, CA, USA).

### ELISA detection of SDF-1α

The NRVCs were cultured with various concentrations of Ex-4 (0–20 nm/l) for 24 h at 37°C and the expression of SDF-1α in the culture supernatant was measured using an ELISA kit (cat. no. R6782; Biotang, Inc., Lexington, MA, USA). The sample (50 *μ*l culture supernatant) was added into 50 *μ*l dilution buffer (phosphate buffer solution plus 0.05% Tween 20, pH 7.2–7.4; Sigma-Aldrich) in each well and it was washed twice. Subsequently, the detection antibody (anti-rat SDF-1α polyclonal antibodies, 2 μ*g/*ml, cat. no. ab9797; Abcam) was added and it was incubated for 1 h at room temperature. The substrate and stop solutions were then added into each well. Immediately following this, the optical density of each well was determined using the microplate reader set to a wavelength of 450 nm (Epoch 2; BioTek Instruments, Inc.).

### RNA extraction and reverse transcription quantitative polymerase chain reaction (RT-qPCR)

Total RNA was extracted from the cells using TRIzol® reagent (Invitrogen Life Technologies, Carlsbad, CA, USA) and was reverse transcribed into a total of 1 *μ*l (60 ng/*μ*l) cDNA using a One-Step RT-PCR kit (TransGen Biotech Co., Ltd., Beijing, China), according to the manufacturer’s instructions. Quantification of gene expression was performed using an ABI PRISM 7500 Sequence Detection system (Applied Biosystems Life Technologies, Foster City, CA) with SYBR® Green (TransGen Biotech Co., Ltd.). The relative mRNA expression levels of SDF-1α and CXCR4 were normalized to that of β-actin using the 2^−ΔΔCT^ method (20). The following primer sequences were used (Shanghai GenePharma Co., Ltd., Shanghai, China): Rat CXCR4, forward 5′-GCTGAGGAGCATGACAGACA-3′ and reverse 5′-GAT GAAGGCCAGGATGAGAA-3′ (21); rat SDF-1α, forward 5′-CTGTTGTGCTTACTTGTTTAAGGCTTTGTC-3′ and reverse 5′-GACGCCAAGGTCGTCGGT-3′ (22) and rat β-actin, forward 5′-GCTACAGCTTCACCACCACA-3′ and reverse 5′-GCCATCTCTTGCTCGAAGTC-3′. The cycling conditions were as follows: 95°C for 10 min, followed by 40 cycles of 95°C for 15 sec and 72°C for 35 sec, for telomere PCR. The experiments were repeated three times with triplicates of each sample.

### Cell chemotaxis assay

The migration of the ADSCs was evaluated using a 24-well Transwell plate with an 8 *μ*m pore size (Corning, Inc., Corning, NY, USA). Briefly, 2 ml normal NRVC-CM (Nor-CM) and 2 ml NRVC-CM induced with Ex-4 (Ex-4-CM) were collected and placed in the lower chamber of the plate and 10^5^ ADSCs, with or without the aforementioned treatment of Ex-4, were added to the upper chamber in serum-free DMEM. The chemotaxis chambers were then incubated for 12 h at 37°C, followed by removal of the non-migrating cells from the upper chamber and fixing of the migrated cells in methanol for 15 min at room temperature prior to staining with 0.05% crystal violet for 15 min at room temperature in the dark. To quantify the levels of chemotaxis, the number of cells that had migrated through to the underside of the insert membranes were counted in at least five randomly selected fields using a BX51 microscope (magnification, ×100; Olympus Corp.). The data were expressed as the ratio of the experimental samples to the control samples × 100.

### Pre-treatment of reagents

Prior to the western blot analysis and migration assay, the cardiomyocytes were pretreated with or without PI3K inhibitor (LY294002; 20 *μ*m/l; Cell Signaling Technology, Inc., Danvers, MA, USA) for 2 h prior to treatment with Ex-4 (20 nm/l) for 24 h. Following treatment, the conditioned medium was collected and used for subsequent experiments. In addition, prior to western blotting and migration assay, the ADSCs were incubated with or without either LY294002 (30 *μ*m/l) for 2 h or an SDF-1α/CXCR4 cascade antagonist (AMD3100; Abcam; 5 *μ*g/ml) (23) for 1 h, prior to treatment with Ex-4 (20 nm/l) for 24 h.

### Statistical analysis

SPSS for Windows version 15.0 (SPSS Inc., Chicago, IL, USA) was used for statistical analyses. The data are expressed as the mean ± standard deviation. One-way analysis of variance was used to determine statistical significance. P<0.05 was considered to indicate a statistically significant difference.

## Results

### Morphological characterization of the ADSCs and NRVCs

**Figure 1:**
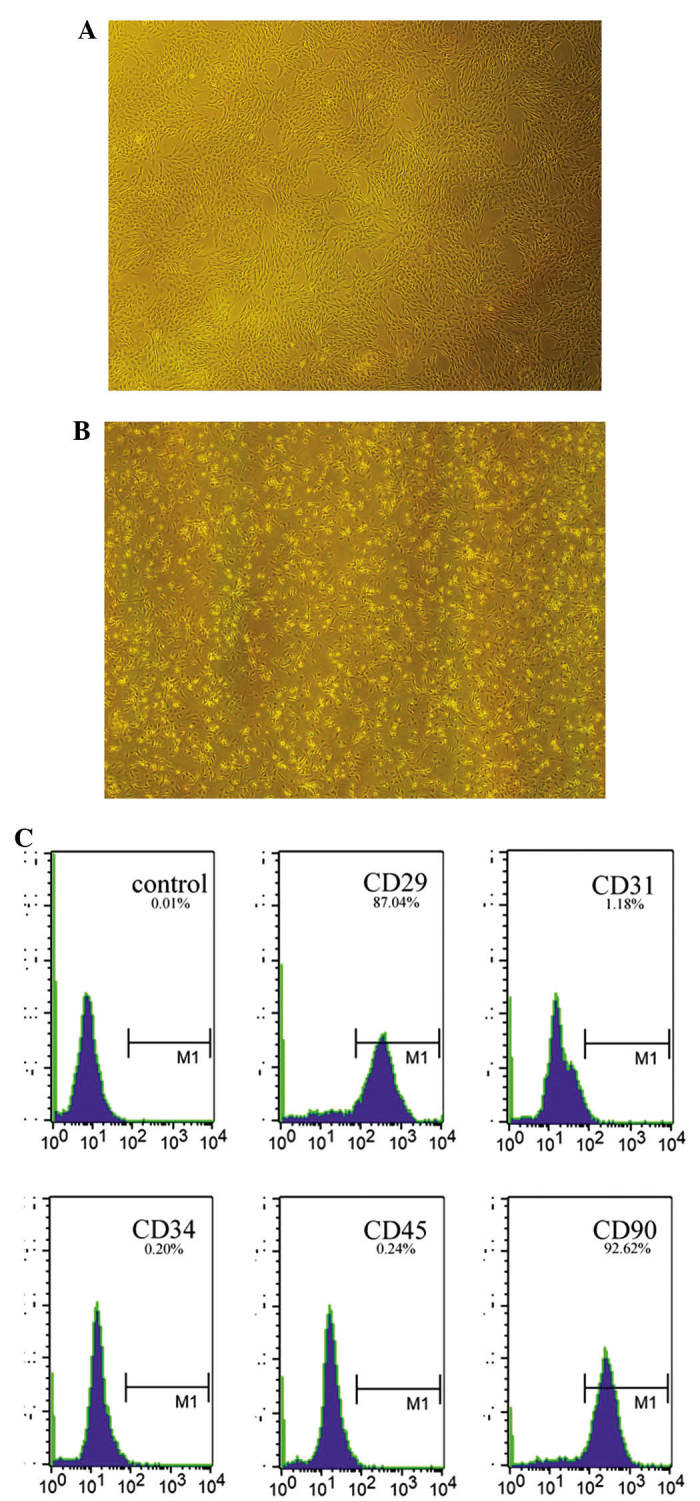
Characterization of ADSCs and NRVCs. (A) Isolated ADSCs exhibited a fibroblast-like shape (magnification, ×40). (B) Morphological characterization of cardiomyocytes (magnification, ×40). (C) Flow cytometry analysis for the phenotypic characterization of ADSCs. ADSC, adipose-derived stem cell; NRVC, neonatal rat ventricular cardiomyocyte; M1, positive range performed by the BD FACSCalibur according to the control groups.

The ADSCs cultured in medium exhibited a spindle-shaped or fibroblast-like morphology (Fig. 1A). After 48 h in culture, the cardiomyocytes were confluent and beating spontaneously (Fig. 1B). In addition, flow cytometry revealed that the ADSCs were positive for the CD29 and CD90 mesenchymal stem cell markers, but were negative for the CD45 and CD34 hematopoietic lineage markers and the CD31 endothelial marker (Fig. 1C). These findings are concordant with those of a previous study (24).

### Effects of Ex-4 on the proliferation of ADSCs

**Figure 2:**
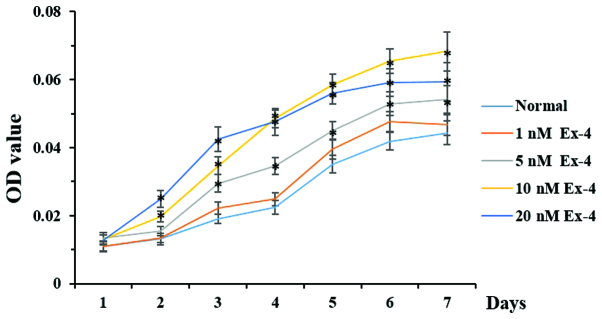
Growth curve demonstrating the effects of Ex-4 on the proliferation of ADSCs. The optimum concentration of Ex-4 for the growth of ADSCs was 10 nm. Values are expressed as the mean ± standard deviation. *P<0.01, as compared with the normal group. Ex-4, exendin-4; ADSC, adipose-derived stem cell; OD, optical density.

A dose-dependent increase in the proliferative capacity of the ADSCs was observed following treatment with Ex-4 (Fig. 2). The optimal concentration of Ex-4 in stimulating the proliferation of ADSCs was 10 nm. The proliferation of the ADSCs was markedly higher in the cells treated with 20 nm Ex-4 compared with the other groups between 1 and 4 days, but was lower compared with the 10 nm group between 4 and 7 days. Furthermore, no significant differences were observed in the proliferative ability of the cells between the 1 nm group and the normal control group; and, during the first 24 h, no statistical differences were observed between any of the groups.

### Ex-4 upregulates the expression of CXCR4 in ADSCs and the production of SDF-1α in NRVCs

**Figure 3:**
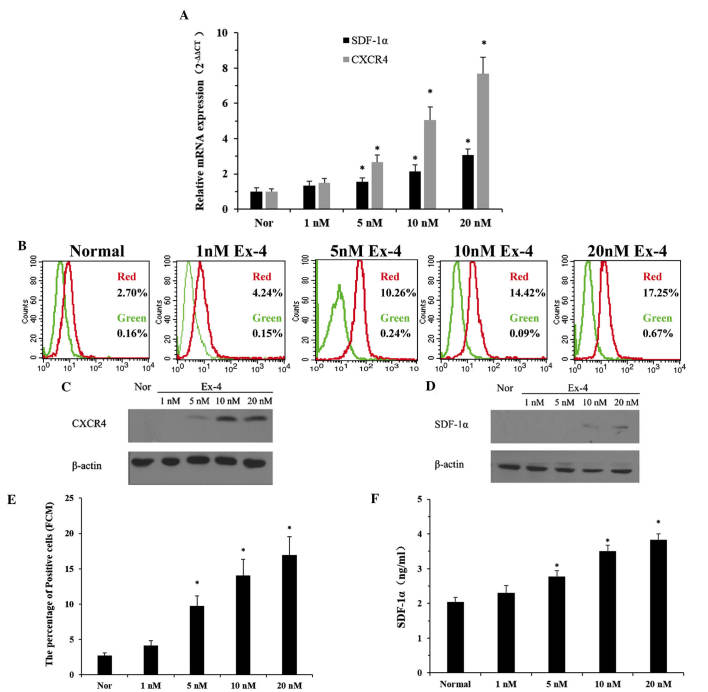
Effects of Ex-4 on the expression levels of CXCR4 in ADSCs and the secretion of SDF-1α in NRVCs. (A) Reverse transcription quantitative polymerase chain reaction was used to determine the mRNA expression levels of CXCR4 and SDF-1α in the ADSCs and NRVCs, respectively, following treatment with various concentrations of Ex-4 (0–20 nm/l) for 24 h. (B) Flow cytometry was used to analyze the levels of CXCR4 in the ADSCs at passage 4 following treatment with Ex-4. Red, ADSCs treated with various concentrations of Ex-4 for 24 h and then incubated with FITC-labeled CXCR4; green, ADSCs treated with various concentrations of Ex-4 for 24 h and then incubated with FITC-labeled immunoglobulin G.Western blot analysis was performed to detect the protein expression levels of (C) CXCR4 and (D) SDF-1α. Flow cytometric analysis of the (E) percentage of CXCR4 positive cells in the ADSCs at passage 4 and the (F) concentration of SDF-1α in the supernatant of culture medium obtained from NRVCs. Values are expressed as the mean ± standard deviation. *P<0.05, vs. normal group. ADSC, adipose-derived stem cell; NRVC, neonatal rat ventricular cardiomyocyte; Ex-4, exendin-4; CXCR4, CXC chemokine receptor 4; SDF-1α, stromal cell-derived factor-1α; Nor, normal control (0 nm/l Ex-4).

Since no significant difference was observed in the proliferative ability of the cells following treatment with or without Ex-4 for 24 h, the cells were treated with Ex-4 for 24 h in the subsequent experiments to exclude the effects of proliferation on the results. The ADSCs were treated with various concentrations (0–20 nm/l) of Ex-4 for 24 h, following which the mRNA expression levels of CXCR4 were analyzed by RT-qPCR. Treatment with Ex-4 caused a dose-dependent increase in the mRNA expression levels of CXCR4, which was highest in the cells treated with 20 nm Ex-4 (Fig. 3A). Furthermore, to confirm whether the upregulation of CXCR4 mRNA resulted in increased protein translation, the protein expression levels were measured on the cell surface (Fig. 3B) and intracellularly (Fig. 3C) and by western blot analysis and flow cytometry, respectively. Consistent with the enhanced mRNA expression levels of CXCR4, the western blot analysis revealed that the ADSCs also exhibited higher protein expression levels of CXCR4 following treatment with Ex-4 (Fig. 3C). These results suggested that the treatment of ADSCs with Ex-4 enhanced the mRNA and protein expression of CXCR4.

The present study also aimed to determine whether treatment with Ex-4 upregulated the expression of SDF-1α in the NRVCs. The cells were incubated with Ex-4 (0–20 nm/l) for 24 h and the mRNA and protein expression levels of SDF-1α were then analyzed by RT-qPCR, western blot analysis and ELISA. The mRNA expression levels of SDF-1α, relative to the levels of a constitutively expressed control gene, were ~three times higher in the 20 nm group compared with the normal group (Fig. 3A). The protein expression levels exhibited a similar pattern to the mRNA expression levels, as determined by western blotting (Fig. 3D). In addition, flow cytometric analysis of the two groups of cells revealed an increased number of CXCR4-positive cells in the P_4_ ADSCs compared with the control group, peaking at >6-fold higher (16.92±2.59% in the 20 nm group, vs 2.73±0.35% in the normal group (Fig. 3E) and increased protein expression levels of autocrine SDF-1α following incubation of the NRVCs with Ex-4 (Fig. 3F). The concentration of SDF-1α in the supernatant of the culture medium was higher in the cells treated with 20 nm Ex-4 compared with the normal group (3.8±0.19 vs. 2.03±0.14 ng/ml; P<0.05; n=3). However, no statistical difference was observed between the cells treated with 1 nm Ex-4 and the normal group.

### Ex-4 promotes the migration of ADSCs to NRVC-CM

**Figure 4:**
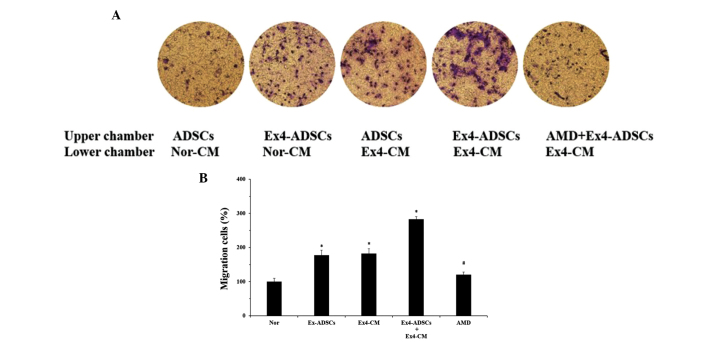
Effects of Ex-4 on the migration of ADSCs to NRVC-CM. Ex-4-CM and Nor-CM were obtained from the NRVCs, which were added to the lower chamber of a Transwell plate. ADSCs treated with Ex-4 (Ex-ADSCs), untreated ADSCs, and Ex-ADSCs pretreated with AMD3100 (5 *μ*g/ml) were placed in the upper chamber. Following a 12 h incubation at 37°C, the non-migrating ADSCs were removed and the migrated cells were stained with 0.05% crystal-violet, followed by observation under a fluorescence microscope. The data are expressed as the ratio of the experimental samples to the normal samples which were designated as 100%. (A) Treatment with Ex-4 increased the migration of ADSCs to NRVC-CM and this effect was reversed by pretreatment with AMD3100 (magnification, ×100). (B) Relative percentage of migrated cells in the experimental groups compared with the normal group. Results are representative of three separate experiments. Values are expressed as the mean ± standard deviation.*P<0.05, vs. normal group; ^#^P<0.05, compared with the Ex-ADSCs and Ex-4-CM groups. ADSC, adipose-derived stem cell; NRVC, neonatal rat ventricular cardiomyocte; CM, conditioned medium; Ex-4, exendin-4; AMD. AMD3100; Nor, normal control (Nor-CM).

A chemotaxis assay was performed in order to confirm whether alterations in the expression levels of SDF-1α and CXCR4 in ADSCs and NRVC-CM, following treatment with Ex-4, promotes chemoattraction. Treatment of ADSCs with 20 nm Ex-4 for 24 h resulted in a marked increase in the migration of ADSCs in response to Nor-CM (Fig. 4). In addition, the ADSCs were more attracted to Ex-4-CM than Nor-CM. Notably, in the presence of Ex-4-CM, the Ex-4-ADSCs had the highest migratory response. These data indicated that Ex-4 improved the migration of ADSCs and enhanced the chemotactic abilities of the NRVC-CM. To confirm whether Ex-4 increased the migration of ADSCs to NRVC-CM via upregulation of the SDF-1α/CXCR4 cascade, the ADSCs were pretreated with AMD3100 (5 *μ*g/ml), a SDF-1α/CXCR4 cascade antagonist, which inhibits the binding of SDF-1α to CXCR4 As expected, the increased chemotactic response of the Ex-4-ADSCs to the Ex-4-CM was markedly inhibited by pretreatment with AMD3100 (Fig. 4). These results suggested that Ex-4-mediated cell migration was dependent on the interaction between SDF-1α and CXCR4 and that the increased expression of CXCR4 in the Ex-4-ADSCs and of SDF-1α in the Ex-4-CM were responsible for the upregulation in ADSC migration.

### Ex-4-mediated upregulation of the SDF-1α/CXCR4 cascade is dependent on the PI3K/Akt pathway in ADSCs and NRVCs

**Figure 5:**
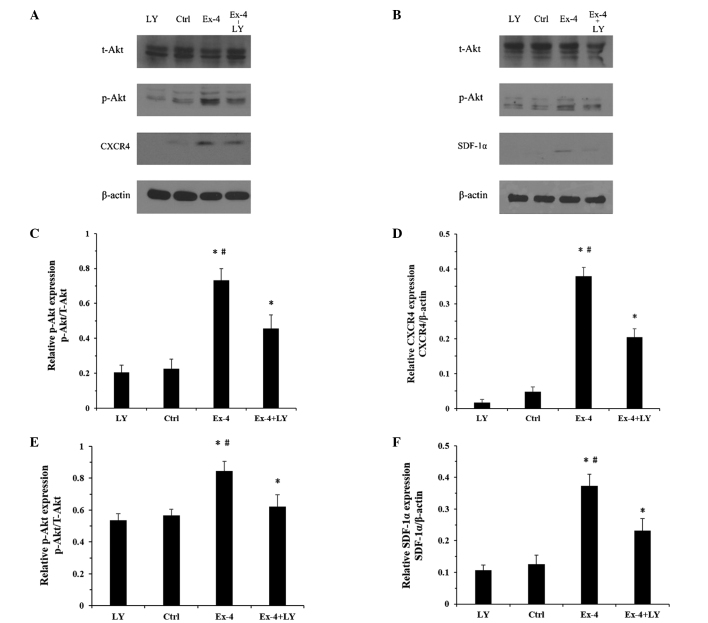
Western blot analysis of the protein expression levels of t-Akt, p-Akt, CXCR4 and SDF-1α in the ADSCs and NRVCs treated with Ex-4 and/or LY. β-actin was used as an internal reference protein. (A) Changes in the protein expression levels in ADSCs; (B) Changes in the protein expression levels in the NRVCs. (C and D) Relative changes in the protein expression levels of p-Akt and CXCR4 in ADSCs. (E and F) Relative changes in the protein expression levels of p-Akt and SDF-α in NRVCs. Values are expressed as the mean ± standard deviation. *P<0.05, vs. ctrl. ADSC, adipose-derived stem cells; NRVC, neonatal rat ventricular cardiomyocyte; Ex-4, exendin-4; CXCR4, CXC chemokine receptor 4; SDF-1α, stromal cell-derived factor-1α; t-Akt, total Akt; p-Akt, phosphorylated Akt; LY, LY294002; ctrl, control (untreated).

Since the increased secretion of SDF-1α and expression of CXCR4 is regulated by the PI3K/Akt pathway (25) and Akt is a downstream target of Ex-4 (26), the present study hypothesized that the PI3K/Akt pathway may contribute to the Ex-4-mediated expression of SDF-1α and CXCR4 in the ADSCs and NRVCs, respectively. To confirm this hypothesis, the expression levels of p-Akt were examined in the two cell lines following treatment with Ex-4. Treatment with Ex-4 increased the protein expression levels of p-Akt in the two cell types (Fig. 5A and B). Furthermore, a PI3K/Akt pathway inhibitor was used to determine the role of the PI3K/Akt pathway on the expression of SDF-1α and CXCR4 in the ADSCs and NRVCs, respectively. The cells were pretreated for 2 h with the LY294002 PI3K/Akt inhibitor, followed by treatment with Ex-4 (20 nm/l) for 24 h. The protein expression levels of p-Akt were markedly inhibited following treatment with LY-294002 in the ADSCs and NRVCs (Fig. 5A and B) and treatment with the PI3K/Akt inhibitor significantly inhibited the Ex-4-mediated upregulation of SDF-1α and CXCR4 in the ADSCs and NRVCs, respectively (Fig. 5CF). These results confirmed that Ex-4 induced an upregulation in the expression levels of SDF-1α and CXCR4 via the PI3K/Akt pathway.

### PI3K/Akt pathway is involved in the migratory response of ADSCs to NRVC-CM

**Figure 6:**
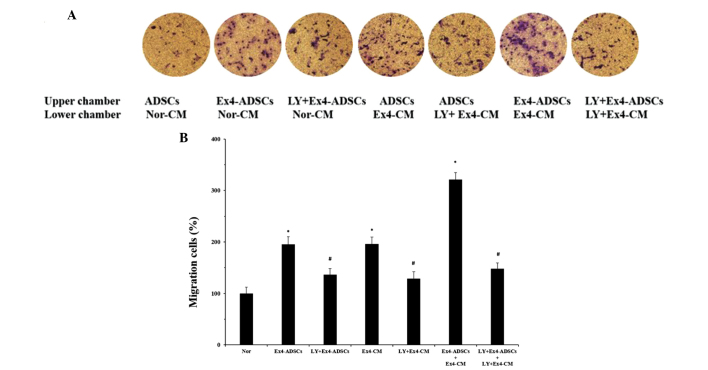
Effects of the PI3K/Akt pathway on the migration of ADSCs to NRVC-CM. The NRVCs were treated with or without the LY294002 for 2 h, followed by treatment with Ex-4 (20 nm) for 24 h. Ex-4-CM and LY+Ex-4-CM, were collected from the NRVCs and added to the lower chamber of a Transwell plate. The same treatment were applied to the ADSCs and the Ex-4-ADSCs and LY+Ex-4-ADSCs were added to the upper chamber. Untreated ADSCs and Nor-CM were defined as the Nor groups. After 12 h incubation at 37°C, the migrated cells were observed in at least five randomly selected fields. (A) PI3K/Akt pathway was involved in the Ex-4-mediated migration of ADSCs to NRVC-CM, since pretreatment with LY294002 partially inhibited the heightened migratory response induced by Ex-4 (magnification, ×100). (B) Data are expressed as the ratio of migrated cells in the experimental groups relative to the Nor group. Results are representative of three separate experiments. Values are expressed as the mean ± standard deviation.*P<0.05, vs. control group; ^#^P<0.05, vs. Ex-4 treatment groups, including Ex4-ADSCs, Ex4-CM, and Ex4-ADSCs+Ex4-CM. ADSC, adipose-derived stem cell; NRVC, neonatal rat ventricular cardiomyocyte; CM, conditioned medium; Ex-4, exendin-4; PI3K, phosphoinositide 3-kinase; LY, LY294002; Nor, normal control group.

The present study also aimed to identify the potential role of the PI3K/Akt pathway on the migration of the ADSCs to the NRVC-CM. The NRVCs were pretreated with or without the LY294002 PI3K/Akt inhibitor (20 *μ*m/ml) and then treated with Ex-4 (20 nm/l) for 24 h. The NRVC-CM were collected and a Transwell assay was performed. Fewer ADSCs translocated through the membrane following pretreatment of the NRVCs with LY294002 (Fig. 6). In addition, the ADSCs were pretreated with LY294002 (30 *μ*m/ml) and, as expected, the number of migratory ADSCs decreased. When the NRVCs and ADSCs were pretreated with LY294002 for 2 h, followed by treatment with Ex-4 for 24 h, the migratory response of the ADSCs to the NRVC-CM was minimal. These results indicated that inhibition of the PI3K/Akt pathway partially impaired the effects of Ex-4 on the migration of the ADSCs to the NRVC-CM.

## Discussion

Stem cell transplantation has recently emerged as a promising tool for the treatment of AMI and ADSCs appear to be a suitable candidate for stem cell therapy. However, despite the improved cardiac function and reduced infarct size observed following injection of ADSCs, the clinical benefits and long-term outcomes remain under debate (27–29). The major obstacle in ADSC therapy is the washout of transplanted cells from the heart (30). The magnitude of cell washout may depend on the presence of cell traffcking and/or homing factors in transplanted cells and the heart. The SDF-1α/CXCR4 cascade has previously been identified as a key factor in the recruitment of stem cells to areas of injured tissue in multiple organ systems (31–33), which is fundamental in stem cell therapy following AMI (34). Briefly, on binding to CXCR4, SDF-1α induces the mobilization of calcium, decreases levels of cyclic AMP within the cells and activates several signaling pathways (35). Ultimately, SDF-1α-bound CXCR4 causes cytoskeletal rearrangement, adhesion to endothelial cells and the polarized migration of cells to specific organs (36,37). Although SDF-1α is predominantly secreted by endothelial cells and upregulated in areas of infarction in short periods of time (9,31), CXCR4 is expressed only in the early passage of ADSCs (38). Therefore, several strategies have been attempted to augment or stabilize the expression of SDF-1α and CXCR4, including genetic modification and altering culture conditions (14,39,40). However, the high cost and adverse side effects of these strategies have limited their application. The present study used a novel drug, Ex-4, to alter the SDF-1α/CXCR4 cascade in cardiomyocytes and ADSCs, and its effects on the SDFA-1α/CXCR4 cascade and migration of ADSCs were observed. Ex-4 is an antidiabetic agent, which reduces hyperglycemia through increased glucose-dependent insulin secretion, glucagon suppression, delayed gastric emptying and appetite suppression (41). It has been demonstrated that Ex-4 is important for cardiopotection, including the reduction in infarction size, improvement in left ventricular ejection fraction (42) and reversion of cardiac remodeling (43). The present study revealed that treatment with Ex-4 significantly increased the secretion of SDF-1α from cardiomyocytes and increased the number of CXCR4^+^ ADSCs. Furthermore, an increased number of ADSCs migrated to the NRVC-CM following treatment with Ex-4. These results demonstrated that Ex-4 was capable of upregulating the SDF-1α/CXCR4 cascade and the chemotaxis of ADSCs to NRVC-CM. Therefore, Ex-4 may be a mediator with an important role in advancing the migration of ADSCs. It was previously demonstrated that SDF-1α alone has cardioprotective effects without the involvement of stem cells (44,45). Following direct administration of SDF-1α into the left ventricle cavity *in vivo*, SDF-1α activates the reperfusion injury signaling kinase pathway in cardiomyocytes, resulting in the recruitment of the anti-apoptotic kinases, extracellular signal-regulated kinase (44), Akt (44) and signal transducer and activator of transcription 3 (45). This promotes an anti-apoptotic response, which confers protection against ischemia/reperfusion damage, as part of the intrinsic repair mechanism following AMI (46). The present study demonstrated that Ex-4 improved the autocrine functions of SDF-1α in cardiac myocytes, which enhanced the endogenous repair system to preserve cardiac performance following AMI. Further studies are required to confirm whether the upregulation of SDF-1α production in cardiomyocytes *in vivo* is sufficient to ameliorate cardiac function following AMI.

Li *et al* (9) previously demonstrated that the expression of CXCR4 declined between 51.4% in P_0_ ADSCs and 2.54% in P_3_ ADSCs *in vitro*, which may affect the homing and reparative potential of ADSCs, impairing the efficacy of ADSC-based therapy on ischemic or injured tissue (9). The results of the present study demonstrated that P_4_ ADSCs with a weak migratory response contained only 2.73% CXCR4^+^ cells, which was consistent with the results of the previous study (9). However, following treatment with Ex-4, the expression of CXCR4 in the P_4_ ADSCs increased in a dose-dependent manner, resulting in a higher number of ADSCs in the lower Transwell chamber. These results indicated that exposure of ADSCs to Ex-4 may increase the expression of CXCR4, functionally contributing to the increased migration of ADSCs. This suggested that Ex-4 may be used as an adjuvant to optimize the phenotype and subsequent function of ADSCs *in vitro*, however, whether the change in phenotype and function of ADSCs is associated with the increased efficiency of cell-based therapy requires further investigation.

It may be hypothesized that increased expression of the SDF-1α/CXCR4 cascade may explain the modulatory role of Ex-4 in the migration of ADSCs; and the increased expression of SDF-1α/CXCR4 may be responsible for upregulating the migration of ADSCs to NRVC-CM. To assess this hypothesis, the ADSCs were pretreated with AMD3100, a specific antagonist of the SDF-1α/CXCR4 cascade. As expected, treatment with AMD3100 inhibited the upregulation of Ex-4-induced ADSC migration. These results indicated that the Ex-4-mediated improvement in the chemotaxis of the ADSCs to the NRVC-CM was due to enhancement of the SDF-1α/CXCR4 cascade. These findings were concordant with those of previous studies, which reported that the SDF-1α/CXCR4 cascade contributed to cell mobilization and the homing of hematopoietic and mesenchymal stem cells (45–50). Numerous studies have focused on the benefits of Ex-4 in the treatment of AMI and post-MI, whereas the present study demonstrated the significant role of Ex-4 in enhancing the SDF-1α/CXCR4 cascade and the subsequent migration of ADSCs, which may assist in improving the grafting of stem cells in clinical transplantation. However, the underlying mechanisms remain to be fully elucidated.

Akt is an important mediator of stem cell survival (51), growth and paracrine mechanisms (52), which regulates various biological responses by phosphorylating a number of substrates. Previous studies have demonstrated that the cardioprotective effects of Ex-4 are associated with the activation of the PI3K/Akt signaling pathway (26,53). Coincidentally, the PI3K/Akt signaling pathway has been observed to regulate the secretion of SDF-1α and the expression of CXCR4 (54–57). Therefore, whether Akt is important in the Ex-4-induced expression of SDF-1α/CXCR4 and the upregulation of ADSC migration to NRVC-CM. The results revealed increased expression levels of p-Akt in the two types of cells, with increased expression of SDF-1α/C XCR4 following treatment with Ex-4, whereas in normal untreated groups with low expression levels of p-Akt, the expression of SDF-1α/CXCR4 expression was minimal. However, this effect was inhibited by treatment with the LY294002 PI3K/Akt inhibitor, suggesting the importance of the PI3K/Akt signaling pathway in Ex-4-induced SDF-1α/CXCR4 expression. In addition, treatment with LY294002 reduced the migration of the ADSCs to the NRVC-CM, suggesting that the PI3K/Akt signaling was involved in the process of ADSC migration to the NRVC-CM. These results revealed that the PI3K/Akt-SDF-1α/CXCR4 pathway was essential for the Ex-4-induced migration of ADSCs, suggesting potential therapeutic applications for Ex-4, including the facilitation of stem cell recruitment and homing.

In conclusion, the present study provided novel insight into potential methods to improve the migration of ADSCs and identified several key signaling molecules involved in this process, including PI3K/Akt and SDF-1α/CXCR4. The PI3K/Akt pathway was found to be responsible for the Ex-4-induced upregulation of the SDF-1α/CXCR4 cascade, which represents the final mediator of ADSC migration and, therefore, offers a potential mechanism to maximize the effectiveness of ADSC-based therapy. However, the detailed genetic changes underlying Ex-4, and whether the increased migratory capacity of the ADSCs observed *in vitro* is associated with improved recruitment and homing of transplanted cells *in vivo* requires further investigation.
